# Supplementary material for: Identification of novel therapeutic inhibitors against E6 and E7 oncogenes of HPV-16 associated with cervical cancer
Source: PLoS One. 2025 Oct 27;20(10):e0323595. doi: 10.1371/journal.pone.0323595 (PMC12558539; doi:10.1371/journal.pone.0323595)
Supplement: Attachment — Submitted filename: Response_to_reviewers.docx [file pone.0323595.s002.docx]

**Author‘s Response Sheet**

**Manuscript Title:** Identification of novel therapeutic inhibitors against E6 and E7 oncogenes of HPV-16 associated with cervical cancer

**Manuscript ID:** PONE-D-25-19182

**Journal Requirements:**

| **Sr. No** | **Editor Comments** | **Author Response** |
| --- | --- | --- |
| 1. | Thank you for updating your data availability statement. You note that your data are available within the Supporting Information files, but no such files have been included with your submission. At this time we ask that you please upload your minimal data set as a Supporting Information file, or to a public repository such as Figshare or Dryad. | Thank you for your comment. We have provided raw data for generation of our mentioned results in manuscript |
| 2. | We notice that your revision was submitted on Aug 12 2025, but the manuscript file in your submission's file inventory was uploaded on Apr 10 2025. Please upload the latest version of your revised manuscript as the main article file, with the item type 'Manuscript,' ensuring that it does not contain any tracked changes or highlighting. This will be used in the production process if your manuscript is accepted. | We acknowledge our mistake and load updated file now |
| 3. | Your ethics statement should only appear in the Methods section of your manuscript. If your ethics statement is written in any section besides the Methods, please delete it from any other section. | Respective changes have been added in manuscript file |
